# Halogen‐Free Anisotropic Atomic‐Layer Etching of HfO_2_ at Room Temperature

**DOI:** 10.1002/smsc.202500251

**Published:** 2025-07-22

**Authors:** Shih‐Nan Hsiao, Pak‐Man Yiu, Li‐Chun Chang, Jyh‐Wei Lee, Makoto Sekine, Masaru Hori

**Affiliations:** ^1^ Center for Low‐temperature Plasma Sciences Nagoya University Nagoya 464‐8603 Japan; ^2^ Department of Materials Engineering Ming Chi University of Technology New Taipei City 24301 Taiwan; ^3^ Center for Plasma and Thin Film Technologies Ming Chi University of Technology New Taipei City 24301 Taiwan

**Keywords:** atomic‐layer etching smoothness, HfO_2_ films, nitrogenation, O_2_ plasma, plasma‐assisted atomic‐layer etching

## Abstract

Hafnium(IV) oxide (HfO_2_)‐based materials have attracted substantial interest owing to their outstanding performance in advanced ultrathin semiconductor devices. However, achieving atomic‐level precision and smoothness in HfO_2_ etching remains a major challenge, primarily due to the nonvolatility of reaction products formed with halogen‐based chemicals at room temperature. Herein, a facile cyclic atomic‐layer etching (ALE) process capable of etching HfO_2_ films at room temperature without the use of halogen‐based chemicals is reported. The ALE process consists of a surface nitrogenation step via N^+^‐ion bombardment during N_2_ plasma exposure, followed by O_2_ plasma treatment to remove the surface‐modified layer through the formation of volatile etching byproducts—most likely hafnium nitrates. This process enables precise, subatomic‐level etching of HfO_2_, achieving an etch depth per cycle ranging from 0.23 to 1.07 Å/cycle, depending on the N^+^ ion energy. Additionally, this cyclic ALE method effectively smooths the HfO_2_ surface, yielding a 60% reduction in surface roughness after 20 cycles. Based on the proposed mechanism, this facile ALE process can be extended to other transition metal oxides and offers a sustainable route for fabricating advanced functional oxide‐based devices, without generating corrosive or toxic wastes.

## Graphical abstract

A facile anisotropic atomic‐layer etching process for HfO_2_ films at room temperature without using halogen‐based chemicals is reported. The process comprises a surface nitrogenation step via N^+^ ion bombardment, followed by O_2_ plasma treatment to form volatile etching byproducts. This approach enables subatomic‐level etching precision while simultaneously smoothing the surface.

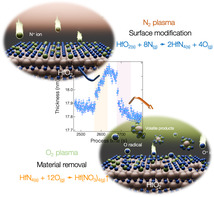

## 1 Introduction

The primary challenge in advancing Si‐based semiconductor devices lies in the difficulty of maintaining continuous dimensional scaling. As critical dimensions shrink to just a few nanometers, performance degradation becomes significant due to excessive heat dissipation and short‐channel effects in transistor or memory devices. Hafnium(IV) oxide (HfO_2_) has emerged as a promising material owing to its excellent electronic properties, which are retained even at such reduced dimensions. These characteristics make HfO_2_ a strong candidate for applications in next‐generation semiconductor devices, including ultrathin gate insulators in 2D material‐based field‐emission transistors and advanced nonvolatile memory devices.^[^ ^1^, ^2^, ^3^ ^]^ However, the development of these advanced semiconductor devices faces a bottleneck in fabricating ultrathin films with atomic smoothness and uniformity. To meet the stringent demands of device scaling, precise atomic‐scale control over both deposition and etching processes—specifically, atomic‐layer deposition (ALD) and atomic‐layer etching (ALE)—has become increasingly essential for accurate pattern transfer.

Atomic‐layer processing refers to a wafer fabrication technique that involves alternating process steps—at least one of them being self‐limiting—thereby enabling atomic‐scale precision and surface smoothness.^[^ ^4^, ^5^ ^]^ ALD has been extensively used in the fabrication of various functional oxide materials, including ultrathin HfO_2_.^[^ ^6^, ^7^, ^8^ ^]^ Its counterpart, ALE, through comparatively slower, has become a critical technique for fabricating advanced semiconductor devices, particularly those featuring complex 3D nanostructures. Thermal ALE, characterized by a lack of directionality, is inherently an isotropic etching process and has been successfully applied to a wide range of materials.^[^ ^9^ ^]^ In contrast, plasma‐enhanced (PE) ALE utilizes energetic species—typically low‐energy ions—to provide the necessary energy for removing surface atoms from materials by forming “volatile products.” Owing to the directionality of these ions, PEALE can be either an anisotropic or nearly isotropic, depending on whether a bias voltage is applied. PEALE has been demonstrated on various materials systems, including Si‐based dielectric materials,^[^ ^10^, ^11^, ^12^, ^13^ ^]^ metal oxides and nitrides,^[^ ^14^, ^15^ ^]^ and 2D materials.^[^ ^16^, ^17^, ^18^ ^]^

In an ideal ALE process, the formation of volatile byproducts is essential. These byproducts, typically generated through fluorination or chlorination using halogen gases, must be volatilized at temperatures determined by the thermodynamic properties of the resulting compounds. HfO_2_ is a well‐known hard‐to‐etch material because its halide product, such as HfF_4_ (HfCl_4_), is highly stable, with very high boiling (melting) temperature of 970 °C (432 °C).^[^ ^19^, ^20^ ^]^ In PEALE of HfO_2_, an Ar beam was first employed by Park et al.^[^ ^21^ ^]^ to remove the hafnium chloride formed during BCl_3_ gas exposure. Subsequently, Lin et al. demonstrated the use of C_4_F_8_/CH_4_ plasmas to deposit hydrofluorocarbon layers, which were then etched via Ar ion bombardment.^[^ ^22^ ^]^ In 2022, the application of Cl_2_/BCl_3_ gas mixtures combined with Ar ion bombardment for the PEALE of HfO_2_ was also reported.^[^ ^23^ ^]^ Additionally, the CF_4_/H_2_/Ar plasmas have been investigated for fluorocarbon‐assisted ALE of HfO_2_,^[^ ^24^ ^]^ utilizing Ar ions for removal. (Detailed process parameters and outcomes of the prior HfO_2_ PEALE studies are summarized in Table S1, Supporting Information). To date, plasma etching of HfO_2_—including conventional reactive ion etching^[^ ^25^, ^26^, ^27^ ^]^—has typically relied on a combination of physical and chemical etching via halogen gases and high‐energy ion bombardment to facilitate to the removal of nonvolatile hafnium halides. However, these physically sputtered byproducts often exhibit low volatility, causing them to adhere to the chamber walls and feature sidewalls. This issue significantly impairs chip‐to‐chip and wafer‐to‐wafer uniformity and thus hinders the scalable integration of complex functional materials such as resistive random‐access memory (RRAM) or ferroelectric random‐access memory (FeRAM)^[^ ^28^, ^29^ ^]^ into cost‐effective mass production. Therefore, thermal ALE is considered a primary method for patterning HfO_2_‐based devices.^[^ ^9^, ^30^, ^31^ ^]^

The rapid growth of the semiconductor industry has underscored the importance of sustainable manufacturing.^[^ ^32^ ^]^ Sustainability considerations include green chemistry and environmental concerns, particularly with regard to greenhouse gas emissions and toxic waste management. However, halogen‐containing chemistries—often highly toxic and/or processing high globe‐warming potential—are still commonly used in etching process, making them unsustainable.^[^ ^33^ ^]^ Therefore, the development of more sustainable etching processes that eliminate the use of halogen‐based chemicals is essential for advancing the fabrication of next‐generation nanoelectronic devices in alignment with sustainable development goals.^[^ ^34^, ^35^ ^]^

**Figure 1:**
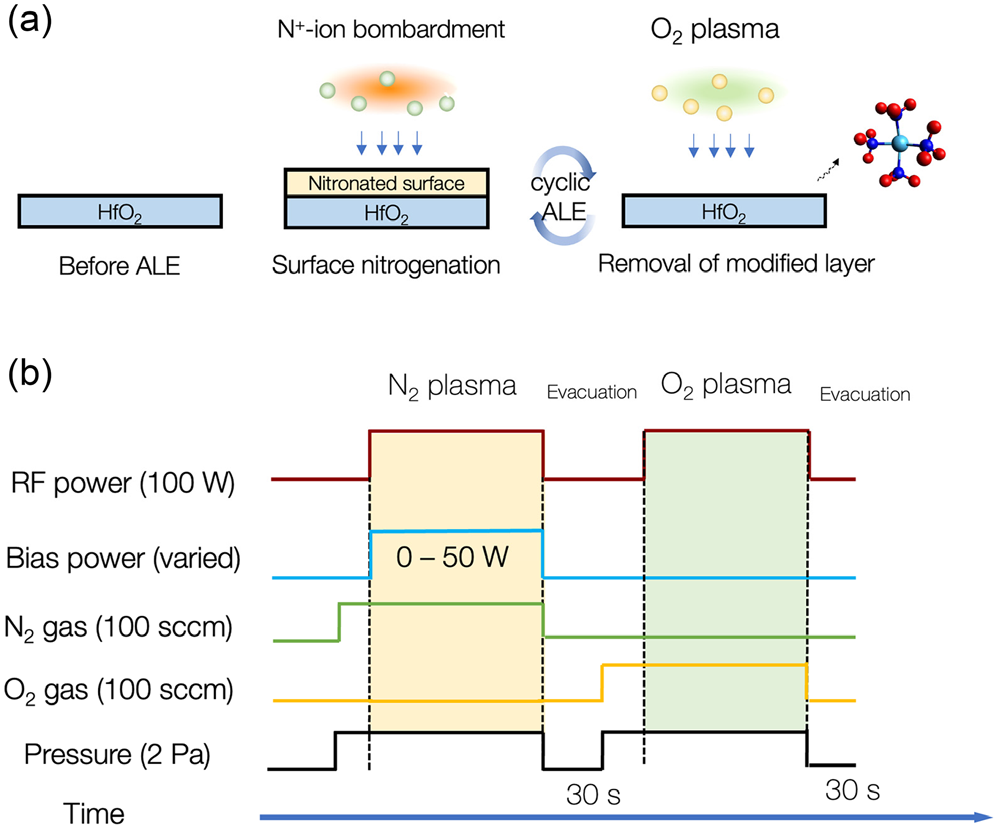
a) Overview of halogen‐free ALE of HfO_2_ at room temperature. b) Parameter sequence in atomic‐layer etching process. The bias power at the bottom electrode during N_2_ plasma discharge was varied from 0 to 50 W. When the bias power was set to 0 W, the substrate remained at a floating potential.

In the present work, we demonstrate a halogen‐free PEALE process for HfO_2_ films at room temperature by alternating N_2_ and O_2_ plasmas, as illustrated in **Figure** **1**a. During the first half‐cycle, N^+^ ion bombardment was used to induce surface nitrogenation of the HfO_2_ film, followed by an O_2_ plasma treatment without an applied bias voltage to remove the surface nitrogenation layer via an observed self‐limiting reaction. As shown in Figure 1b, by adjusting the N^+^ ion energy through the RF power applied to the bottom electrode, the etch depth per cycle (EPC) increased from ≈0.2 to 1.1 Å/cycle. The underlying surface reaction mechanism was elucidated using in situ attenuated total reflection Fourier transformation infrared spectroscopy (ATR‐FTIR) and X‐ray photoelectron spectroscopy (XPS). These analyses consistently revealed the formation of Hf—N bonds through a ligand exchange mechanism, in which nitrogen replaces surface oxygen atoms during the N_2_ plasma exposure. In the subsequent O_2_ plasma half‐cycle, the Hf—N bonds are removed by forming volatile byproducts, most likely, Hf(NO_3_)_*x*_ (1 ≤ *x *≤ 4) species. Furthermore, the process exhibits an ALE smoothing effect, as indicated by the reduction in root‐mean‐square surface roughness from 1.41 to 0.57 nm on the etched HfO_2_ surface.

## 2 Results and Discussion

### 2.1 Real‐Time Thickness Variation of HfO_2_ ALE Process

**Figure 2:**
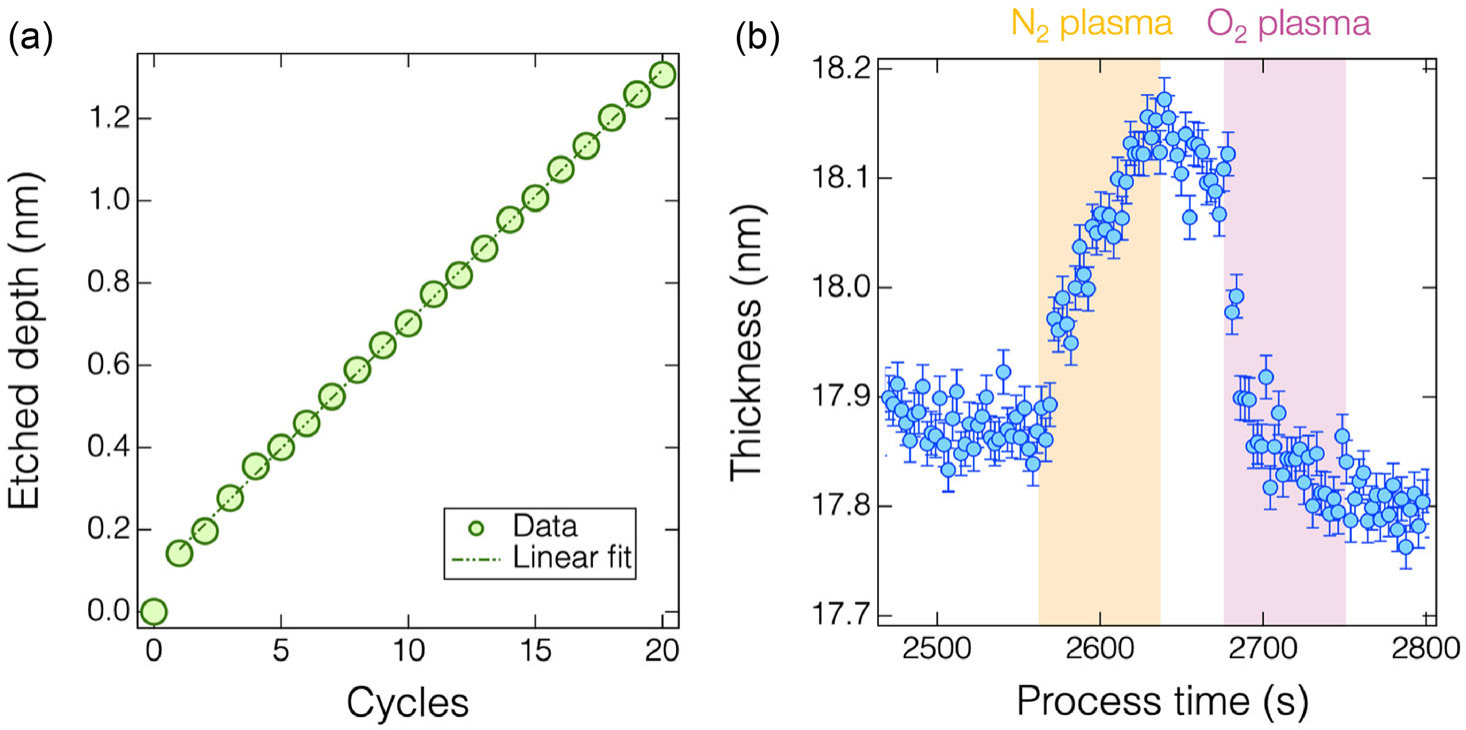
a) Etched depth versus number of cycles during HfO_2_ ALE. b) Thickness changes of HfO_2_ during single cycle highlighted in Figure S1, Supporting Information. The bias power inputs for the N_2_ and O_2_ plasmas were set to 30 and 0 W, respectively.

Figure S1, Supporting Information, shows the typical thickness variation for a HfO_2_ film as a function of the processing time, monitored in real time by in situ spectroscopic ellipsometry over 20 ALE cycles. Each cycle consisted of surface nitrogenation using N_2_ plasma (generated with 100 W for plasma generation and 30 W for bias voltage), followed by a removal step using O_2_ plasma without applying bias voltage. As illustrated in **Figure** **2**a, the etch depth increases linearly with the number of ALE cycles at room temperature, except for the first cycle. This linear trend confirms the stability of the proposed ALE process. The average EPC is ≈0.62 ± 0.02 Å/cycle, indicating subatomic‐level precision, making it potentially suitable for fabricating ultrathin HfO_2_‐based advanced devices.

Figure 2b provides detailed insights into a representative ALE cycle highlighted in Figure S1, Supporting Information. At the beginning of each cycle, a thickness increase of ≈2 Å was observed, attributed to surface nitrogenation induced by N^+^ ion bombardment. It should be noted that the thickness of the HfO_2_ film during the ALE process was monitored using an optical model based on the pristine film. Therefore, the apparent increase in thickness during N_2_ plasma exposure does not accurately reflect the actual thickness of the surface‐modified layer. Upon initiation of the O_2_ plasma, the film thickness decreased, indicating the removal of the surface‐modified layer. In addition, as the oxygen discharge progressed, the etch rate gradually declined and eventually nearly ceased, demonstrating a self‐limiting etching reaction. For more detailed insights into the self‐limiting reactions involved in the process, a representative ALE cycle illustrating the progression to full saturation is presented in Figure S2, Supporting Information.

**Figure 3:**
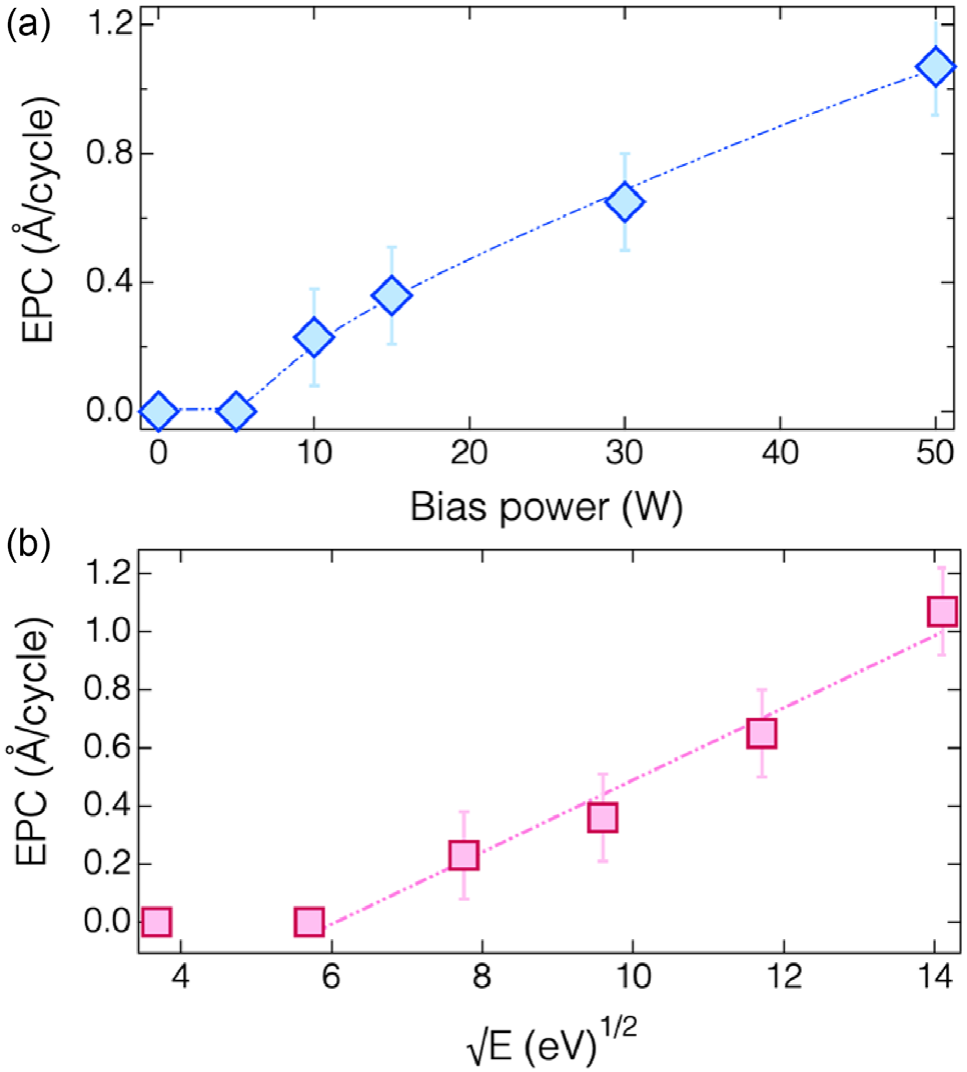
Dependence of EPC on a) bias power input and b) square root of its corresponding ion energy during N_2_ plasma discharge.

To gain a better understanding of the nitrogenation step, the ALE process was performed under various bias power conditions. As illustrated in **Figure** **3**a, the EPC increased from 0 to 1.1 Å/cycleas the bias power input was increased from 0 to 50 W. The obtained EPC values are comparable to or greater than those achieved using halogen‐based ALE processes.^[^ ^21^, ^23^, ^24^ ^]^ This trend suggests that the etching is primarily driven by the incorporation or implantation of N atoms into the HfO_2_ film during N^+^ ion bombardment. To evaluate the energy‐dependent nature of nitrogenation process, the threshold for the nitrogen ligand exchange was estimated on the basis of the collision‐cascade model.^[^ ^36^ ^]^ The relationship between the N^+^ ion energy and the EPC was further examined by measuring the ion energy distribution during N_2_ plasma treatment at various bias power inputs using a retarding field ion energy analyzer^[^ ^37^ ^]^ (see S3, Supporting Information, for details). Figure 3b presents the relationship between the EPC and the average energy of N^+^ ions, clearly demonstrating a typical linear relationship. The threshold energy was determined to be ≈36.6 eV, which is slightly lower than the previously reported sputtering threshold of ≈47 eV for Ar^+^ ions.^[^ ^23^ ^]^

**Figure 4:**
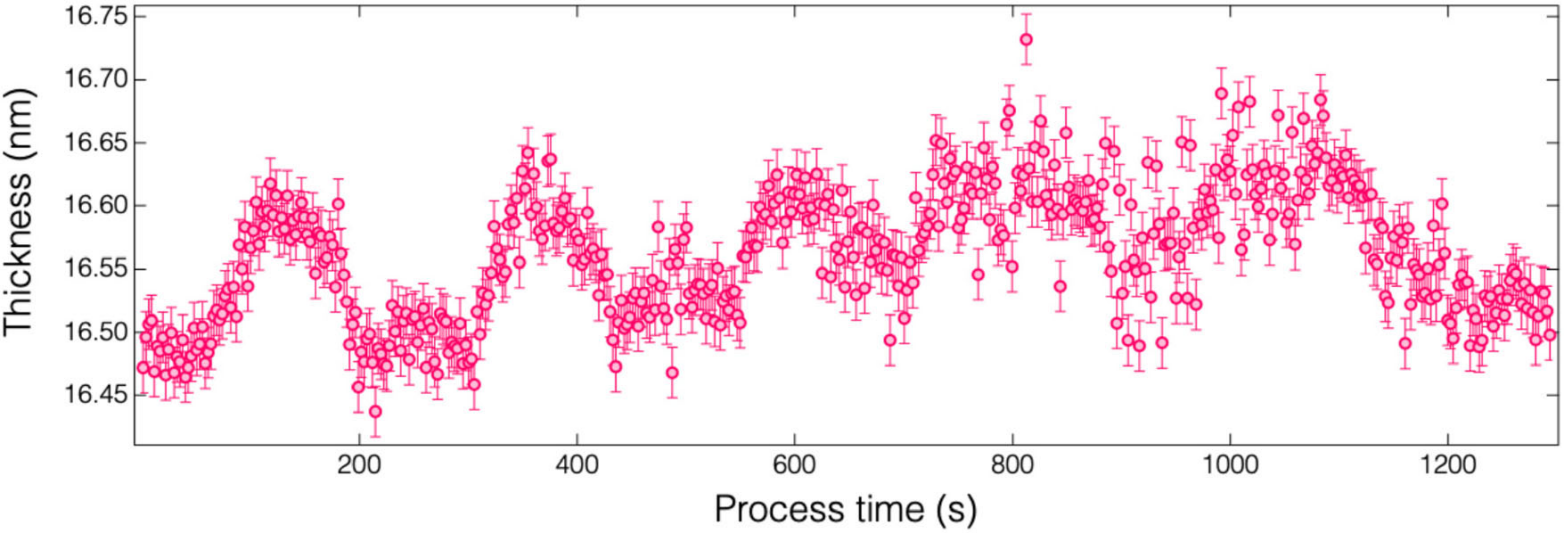
Thickness variation of HfO_2_ film measured in real time using in situ spectroscopic ellipsometry over five cycles of ALE with alternating N_2_ and Ar plasmas.

To investigate the role of O_2_ plasma in the proposed ALE, a pure Ar plasma was employed during the second half‐cycle. **Figure** **4** shows the real‐time thickness variation of the HfO_2_ film during the ALE process, where alternating N_2_ plasma (with a bias power input of 30 W) and Ar plasma were used. The results clearly demonstrate that no etching occurred in the absence of oxygen. Since no bias was applied during the second half‐cycle, the nitrogenated layer—characterized by low volatility—could not be physically sputtered by low‐energy Ar^+^ ions (≈23 eV, as shown in our previous study).^[^ ^37^ ^]^ This finding indicates that volatile byproducts, necessary for material removal, are formed only during O_2_ plasma exposure.

### 2.2 Surface Structure Change during ALE

**Figure 5:**
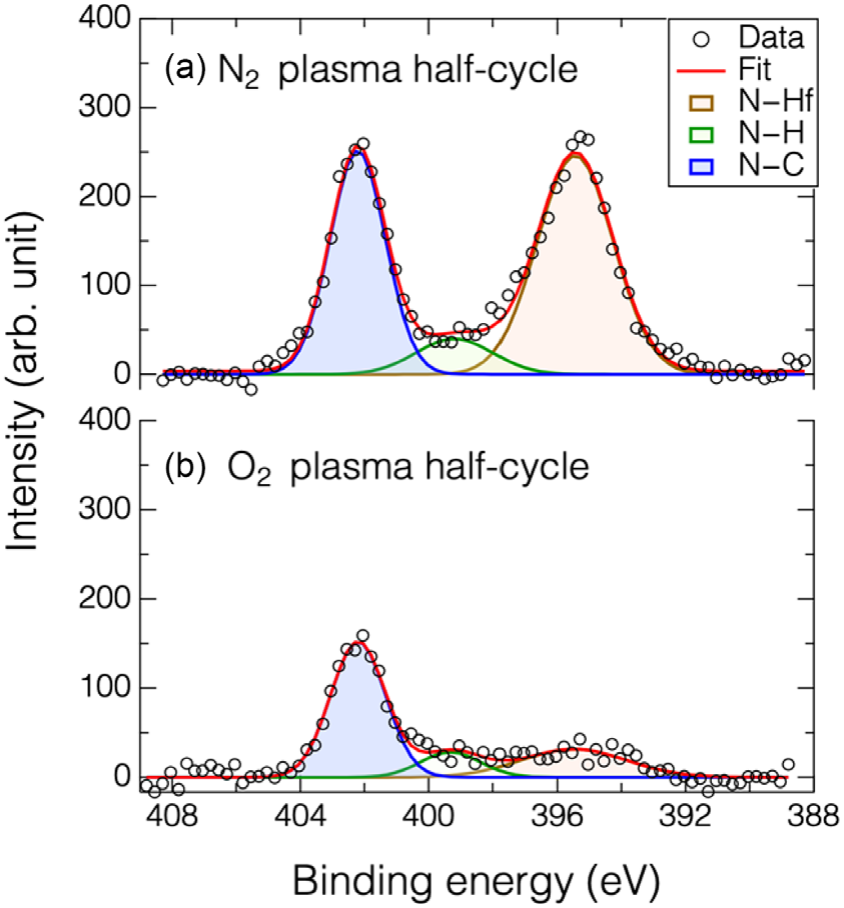
Nitrogen N 1*s* XPS spectra of HfO_2_ film treated with a) N_2_ plasma in first half‐cycle, followed by b) O_2_ plasma in second half‐cycle.

In the XPS spectrum acquired from a pristine sample (see S4, Supporting information), only Hf, O, and C peaks are observed, consistent with previous study.^[^ ^38^ ^]^ For HfO_2_ films prepared by thermal ALD, carbon is a common impurity resulting from the decomposition of metal–organic precursors during deposition.^[^ ^38^, ^39^ ^]^ **Figure** **5** presents the *N* 1*s* XPS spectra, acquired at a take‐off‐angle of 90°, of a HfO_2_ film after sequential N_2_ plasma (with a bias power input of 30 W) and O_2_ plasma treatments. As shown in Figure 5a, two distinct peaks appear in the spectrum after N^+^ ion bombardment, corresponding to Hf—N (≈395 eV) and Hf—CN (≈402 eV) bonds.^[^ ^40^, ^41^ ^]^ These features confirm the incorporation of nitrogen into the HfO_2_ film via ligand exchange, converting surface oxides to nitrides. Following exposure to O_2_ plasma, the overall signal intensity decreased, and the Hf—N component nearly vanished, as illustrated in Figure 5b. This observation clearly indicates that the O_2_ plasma effectively removes the nitrogenated surface layer by forming volatile etching byproducts (see more information regarding Hf 4 *d* and O 1*s* core‐level spectra in Figure S5, Supporting Information).

**Figure 6:**
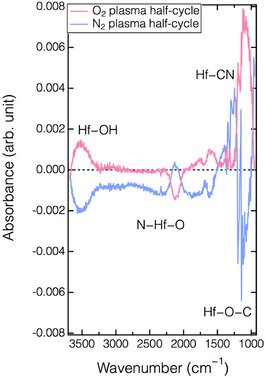
Absorption spectra of HfO_2_ film treated with a) N_2_ plasma in first half‐cycle (blue), followed by b) O_2_ plasma in second half‐cycle (red).

To confirm the surface structural changes during the ALE process, a HfO_2_ film deposited onto a Ge prism using the same deposition process was analyzed in situ via ATR‐FTIR. **Figure** **6** shows the absorbance spectra of the HfO_2_ film throughout an ALE cycle. The reference spectra for the absorbance changes were acquired immediately prior to each process step. After the N^+^ ion bombardment (with a bias power input of 30 W), positive peaks appeared at ≈1250 and 2130 cm^−1^, corresponding to the formation of Hf—N and Hf—CN chemical bonds, respectively.^[^ ^42^, ^43^ ^]^ Conversely, negative peaks at ≈1140 and ≈3550 cm^−1^ were simultaneously observed, indicating a reduction of Hf—OH and Hf—O—C bonds. The Hf—O absorption peaks, which are typically located at ≈400 and ≈500 cm^−1^, were not detected because their positions fall below the wavenumber range for Ge (5500−600 cm^−1^).^[^ ^43^, ^44^ ^]^ These IR spectra indicate that the N^+^ ions can abstract Hf—O bonds, leading to ligand exchange from the oxides to the nitrides, in agreement with the XPS results shown in Figure 5a. During the oxygen plasma half‐cycle, the spectrum reveals opposite behavior, including the removal of Hf—N bonds and the formation of Hf—OH and Hf—O—C chemical bonds. There results also align with the findings from XPS (Figure 6b). Collectively, these findings indicates that oxygen plasma not only removes the Hf—N bonds by forming volatile etching byproducts but also, to some degree, restores the surface structure of the etched HfO_2_. This effect likely resembles the reported ability of O_2_ plasma to reduce oxygen defects in HfO_2_ films prepared by PEALD.^[^ ^45^ ^]^

**Figure 7:**
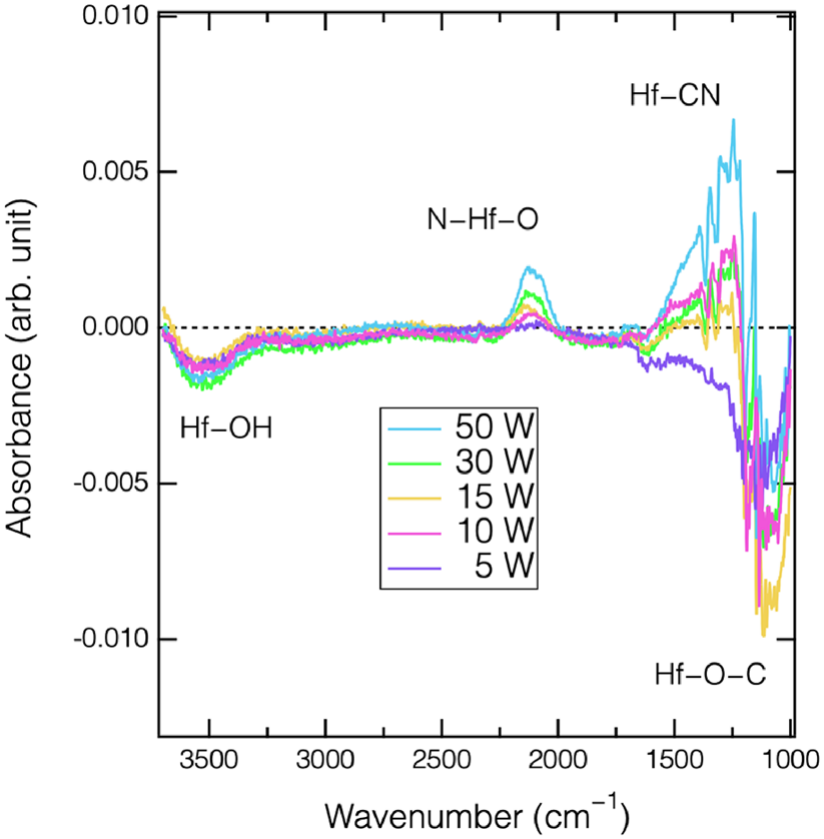
Absorption spectra of HfO_2_ film treated with N_2_ plasma with various bias power inputs ranging from 5 to 50 W.

**Figure 8:**
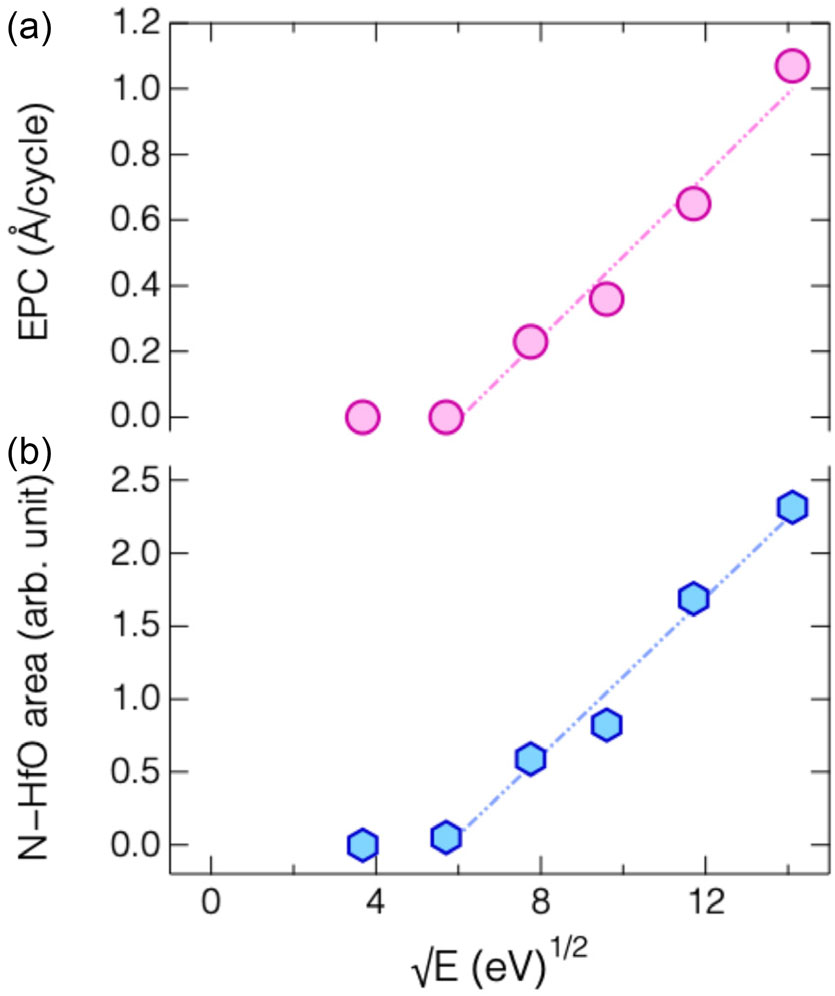
Dependence of a) EPC, reproduced from Figure 3b for comparison, and b) integrated intensity of N—HfO band, as calculated from Figure 7, on square root of N^+^‐ion energy during N_2_ plasma discharge.

**Figure** **7** shows absorbance spectra of the HfO_2_ film following the N_2_ plasma half‐cycle at various bias power inputs. As the bias power increased, the absorbance associated with the N—HfO bands increased during the nitrogenation step increased. The integrated absorbance of these N—HfO was used to estimate the relative concentration of N atoms incorporated into the HfO_2_ surface, based on the assumption that the concentration of the IR‐active species is proportional to the integrated absorbance. **Figure** **8** compares the dependence of both the integrated N—HfO absorbance and the EPC of ALE (reproduced in Figure 2b for comparison) on the root square of the N^+^ ion energy. Remarkably, both quantities exhibit nearly identical trends, implying that the EPC is strongly correlated with the degree of surface nitrogenation induced during N_2_ plasma step. This observation implies that higher‐energy N^+^ ion bombardment facilitates more extensive nitrogen incorporation, thereby enhancing the ALE efficiency and reducing the time needed to reach surface saturation. However, excessive ion energy may also lead to undesired plasma‐induced damage and degradation of film properties.^[^ ^46^ ^]^ Therefore, optimizing the ion energy—either by balancing etching throughput and surface damage or by employing low‐energy plasma techniques^[^ ^18^ ^]^—will be essential for improving the efficiency and reliability of the HfO_2_ ALE process.

### 2.3 Etching Mechanism and ALE Smoothness

**Figure 9:**
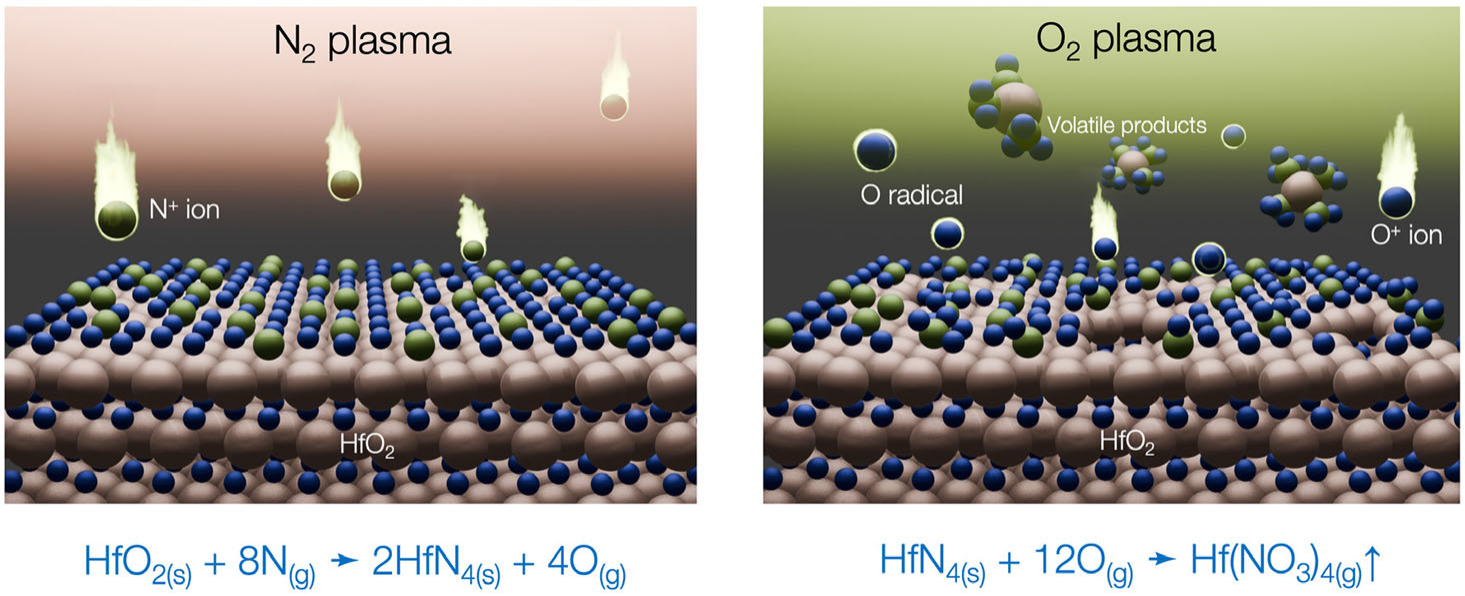
Schematic of etching mechanism for present ALE process.

Based on the etching characteristics and observations from in situ FTIR and XPS, we propose the following etching mechanism for the ALE process, as illustrated in **Figure** **9**. During N_2_ plasma step with a specific bias voltage, the N^+^ ions are accelerated toward the HfO_2_ surface, where ligand exchange occurs, forming Hf—N bonds through direct abstraction of Hf—O bonds by impinging ions and radicals. The complete first half‐cycle reaction can be described as

$${\text{HfO}}_{2\left(s\right)}\;+\;{8N}_{\left(g\right)}\;\to \;{\text{HfN}}_{4\left(s\right)}\;+\;{4O}_{\left(g\right)}$$

Subsequently, low‐energy O ions (at floating potential) are induced to remove the modification layer by forming volatile etching byproducts. The chemical reactivity of oxygen to the nitrogenation surface in this process was confirmed through experiments with Ar plasma, as shown in Figure 4. The complete chemical reactions between the nitrogenated surface and reactive oxygen during the second half‐cycle are most likely as follows:

$${\text{HfN}}_{4(s)}\;+\;12O_{\left(g\right)}\;\to \text{ Hf}{\left({\text{NO}}_{\text{3}}\right)}_{4(g)}$$

where the volatile etching byproducts is expected to be hafnium (IV) nitrate Hf(NO_3_)_4_ or its fragments, i.e., Hf(NO_*x*_)_*y*_, resulting from low‐energy O ion bombardment. Hf(NO_3_)_4_ has a boiling temperature only 75 °C and was commonly used as a precursor for ALD of HfO_2_ film in the early 2000s.^[^ ^47^, ^48^ ^]^ Due to its instability and highly reactive, hafnium nitrate has been replaced by other metal organic compounds and is now rarely used in ALD of HfO_2_.^[^ ^49^ ^]^ One might consider the possibility of organic hafnium molecules, such as tetrakis(dimethylamido)hafnium (Hf(NMe)_2_)_4_, forming during the process due to the presence of carbon impurities in the ALD film. As shown in Figure S6, Supporting Information, the proposed ALE was also successfully demonstrated on carbon‐free HfO_2_ films prepared by radio‐frequency magnetron sputtering (RFMS), further supporting the proposed mechanism. Furthermore, a slightly higher EPC (≈0.81 Å/cycle) was observed in the HfO_2_ films prepared by RFMS compared to those prepared by ALD (≈0.62 Å/cycle), suggesting that the etching characteristics are influenced on presence of impurities in the films. Further investigation is required to fully understand their effect on the ALE mechanism.

**Figure 10:**
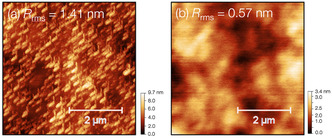
RMS surface roughness image, as analyzed by atomic force microscopy, of HfO_2_ film a) in its pristine state and b) after 20 cycles of ALE.

Surface roughness introduces undesirable variability in material properties that degrades overall device performance, particularly in advanced ultrathin semiconductor devices.^[^ ^50^ ^]^ The smoothing effect is considered one of the key advantages of ALE compared to conventional reactive ion etching.^[^ ^51^, ^52^ ^]^ This effect has been observed and studied across various material systems, such as metals,^[^ ^53^, ^54^ ^]^ oxides and nitrides,^[^ ^55^, ^56^, ^57^ ^]^ and 2D materials.^[^ ^17^ ^]^ As discussed by Sungauer et al., nonvolatile etching products that remain on a surface after etching can lead to increased surface roughness because of the micromasking effect.^[^ ^58^ ^]^ To mitigate this effect, a common strategy is to use high‐ion‐energy bombardment during etching step.^[^ ^23^, ^54^ ^]^ However, these sputtered residues may also adhere to chamber walls, requiring an additional cleaning step to maintain stability of the process.^[^ ^59^ ^]^ As proposed ALE mechanism in this work, an ALE smoothing effect can be expected. **Figure** **10** shows the surface roughness of HfO_2_ film, analyzed by atomic force microscopy, in both the pristine state and after 20 cycles of ALE process. The root‐mean‐square (RMS) roughness was significantly reduced from 1.41 to 0.57 nm, despite the low ion energy of the O_2_ plasma exposure (≈13.5 eV).

## 3 Conclusions

For the first time, halogen‐free atomic‐layer etching of HfO_2_ film was achieved at room temperature. The process consisted of two steps: 1) N^+^ ion bombardment (with applied bias voltage) to induce ligand exchange via surface nitrogenation, followed by 2) O_2_ plasma exposure to remove the surface modification layer. A linear relationship between etched depth and the number of cycles, along with a self‐limiting reaction during O_2_ plasma exposure, was observed. The findings from XPS and in situ FTIR reveal that the ALE progresses through the formation of Hf—N bonds during N^+^ ion bombardment, subsequently followed by their removal via generation of volatile byproducts, i.e., Hf(NO_3_)_4_ molecules or its fragments Hf(NO_x_)_y_, during O_2_ plasma exposure. No etching occurs in the absence of oxygen plasma in the ALE process, highlighting the crucial role of oxygen species on formation of the proposed volatile byproducts. The EPC was found to range from ≈0.23 to 1.07 Å/cycle, proportional to ion energy of N^+^, which influences the degree of nitrogenation, as confirmed by the in situ FTIR. This cyclic ALE method is also applicable for atomic level smoothing, achieving a 60% reduction in surface roughness after 20 ALE cycles. Based on the proposed etching mechanism, this process is expected to be applicable not only to HfO_2_ but also to other transition metal oxides which can form metal nitrate molecules via ligand exchange, such as ziroconium nitrate, vanadium nitrate, and so on. This works highlights the ALE process using halogen‐free plasmas at room temperature as a promising technique for fabrication the advanced functional oxide materials, while aligning with sustainable development goals without generating corrosive and toxic wastes.^[^ ^32^ ^]^

## 4 Experimental Section

4.1

4.1.1

##### Sample Preparation

HfO_2_ films were prepared by thermal ALD and RFMS on Si substrates. A commercial ALD system was used for the atomic‐layer deposited HfO_2_ thin films (Picosun R200, King's College London). The ALD films with a thickness of ≈30 nm were synthesized using tetrakis(dimethylamino)hafnium (TEMAH) and H_2_O as precursors on both Si and Ge prism (20 × 80 × 1 mm^3^, 45° beveled edges) substrates at a substrate temperature *T*_s_ of 225 °C. The pulse durations per cycle for HfO_2_ and H_2_O were 0.8 and 1 s, respectively, resulting in a growth rate of ≈0.1 nm/cycle. For the sample prepared by RFMS, ≈50 nm thick HfO_2_ films were deposited onto a Si substrate using a 76.2 mm diameter HfO_2_ target. The deposition was carried out with Ar plasma with a flow rate of 30 sccm, while the substrate temperature and pressure during deposition were maintained at room temperature and 0.4 Pa, respectively.

##### Cyclic ALE Process

A home‐built capacitively coupled plasma reactor was used for the ALE experiments (Figure S7, Supporting Information). The top electrode was operated at a frequency of 100 MHz for plasma generation, while the substrate was biased using the bottom electrode at a frequency of 2 MHz. The vacuum condition was maintained at a pressure better than 7 × 10^−5^ Pa before conducting experiments. The sample (HfO_2_/Si coupon or Ge prism) was affixed to a Si carrier wafer using a fluorinated grease to improve the thermal conductivity. The substrate temperature was controlled at 20 °C with a circulating coolant system. As shown in Figure S7(a,b), Supporting Information, and Figure 1, a N_2_ gas flow at 100 sccm was introduced through a showerhead top electrode, and the pressure was maintained at 2.0 Pa using an automatic pressure control valve. The power input for the N_2_ plasma discharge was set at 100 W for 1 min, while the bottom electrode bias power varied from 0 to 50 W. Subsequently, the excess N_2_ was evacuated using the pumping system for 30 s, followed by O_2_ plasma exposure with the same conditions as the N_2_ plasma, except that no bias power input was applied.

##### Characterization

The thickness variation during ALE was monitored in real time using a SE system equipped with a Xe light source with a multiwavelength range of 200−1000 nm (M‐2000, J. A. Woollam). Surface structure variation during ALE was analyzed using XPS and in situ FTIR. For the XPS measurements (VG Microtech, MT500 CLAM2), one HfO_2_ film on Si substrate was used for all experiments. Because the ex situ XPS experiments were conducted, the sample was transferred back and forth between the etcher and the XPS chamber, which was located adjacent to the etcher, after venting for each ALE half cycle and XPS measurement step. This approach minimized surface changes or contamination that could occur due to exposure at ambient pressure. The XPS spectra were acquired using an Mg K_α_ source at a take‐off angle of 90°. Before peak deconvolution, the background of the observed XPS spectra was subtracted with a Shirley function. For in situ FTIR measurements, ATR mode was used to analyze surface structure changes during the ALE process. The measurements were performed on the ALD‐prepared HfO_2_/Ge prism sample. The FTIR system (iS50, Thermo Fisher Scientific) utilized an IR light source and a liquid‐nitrogen‐cooled HgCdTe (MCT) detector. The acquired spectra covered a wavenumber range of 700−4000 cm^−1^ with a resolution of 4 cm^−1^. To achieve with high signal‐to‐noise ratio, each data presented in this work was obtained by averaging at least 128 scans. The ion energy of the N_2_ and O_2_ plasma was analyzed with a commercial retarding field energy analyzer (Semion System 500, Impedans Ltd.).

## Conflict of Interest

The authors declare no conflict of interest.

## Supporting information

Supplementary Material

## Data Availability

The data that support the findings of this study are available from the corresponding author upon reasonable request.
