# Supplementary material for: Halogen‐Free Anisotropic Atomic‐Layer Etching of HfO2 at Room Temperature
Source: Small Sci. 2025 Jul 22;5(10):2500251. doi: 10.1002/smsc.202500251 (PMC12499433; doi:10.1002/smsc.202500251)
Supplement: Supplementary file 1 — Supplementary Material [file SMSC-5-2500251-s001.pdf]

Supporting Information

Halogen-Free Anisotropic Atomic Layer Etching of HfO_2 at Room Temperature

Shih-Nan Hsiao[1*], Pak-Man Yiu[2, 3], Li-Chun Chang[2, 3], Jyh-Wei Lee[2, 3], Makoto Sekine[1], and Masaru Hori[1]

[1] Center for Low-temperature Plasma Sciences, Nagoya University, Nagoya, 464-8603, Japan

[2] Department of Materials Engineering, Ming Chi University of Technology, New Taipei City, 24301, Taiwan

[3] Center for Plasma and Thin Film Technologies, Ming Chi University of Technology, New Taipei City, 24301, Taiwan

*Corresponding authors: Shih-Nan Hsiao (hsiao.shih.nan.t8@f.mail.nagoya-u.ac.jp)

Table SI. Summary of the reported plasma-assisted ALE process for HfO_2, including one example of thermal ALE for comparison.

Lead, reference	Surface modification	Material Removal	EPC (Å/cycle)	Substrate temperature (ºC)	ALE smoothness (Å)
Park *et al*. [21]	BCl_3 gas	Ar neutral beam	1.2	N.A.	N.A. (~28)*
Lin *et al*. [22]	C_4F_8/CH_4, CHF_3/CH_4 plasma	Ar plasma	0.5	10	N.A.
De Marneffe *et al*. [23]	Cl_2/BCl_3 gas	Ar plasma	1.25	0	Yes (1.4 to 1)
Kuzmenko *et al*. [24]	$CF_4/H_2/Ar$ plasma	Ar plasma	1.1	20	N.A.
H. Willem**	SF_6 plasma	Ar plasma	5	30	No
Lee *et al*.*** [31]	HF gas	$TiCl_4$ gas	0.59	300	N. A.
This work	N_2 plasma	O_2 plasma	0.23 – 1.07	20	Yes (14 to 5.7)

*The authors did not specify the exact surface roughness value of the sample prior to etching.
**Master thesis, "Anisotropic plasma atomic layer etching of Al_2O_3 and HfO_2 using SF_6 and Ar plasma", Eindhoven University of Technology, 2022.
***Thermal ALE

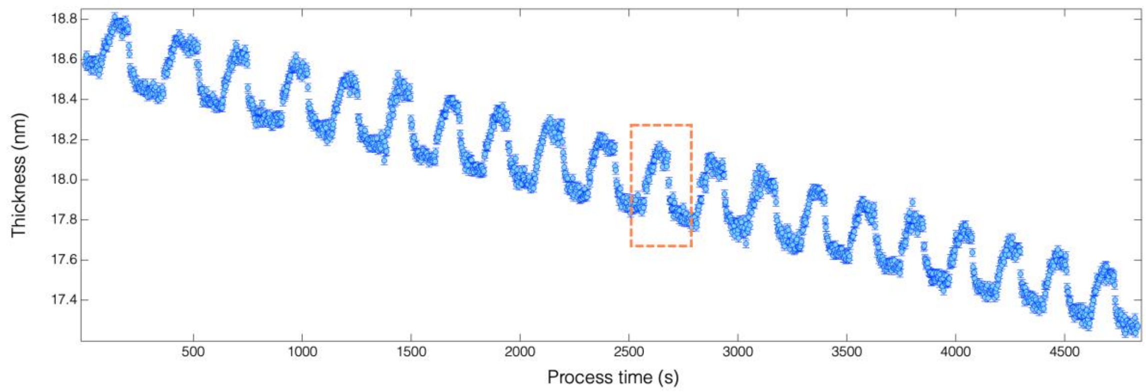

Fig. S1. Thickness variation of HfO_2 film measured in real time over 20 cycles of ALE, as measured using in situ spectroscopic ellipsometry. The RF power for plasma discharges was set to 100 W with a duration of 1 minute. The bias power inputs for the N_2 and O_2 plasmas were set to 30 W and 0 W, respectively. An interval of 30 seconds was allowed for residual gas evacuation between N_2 and O_2 plasma discharge. The pressure during plasma discharges was maintained at 4 Pa.

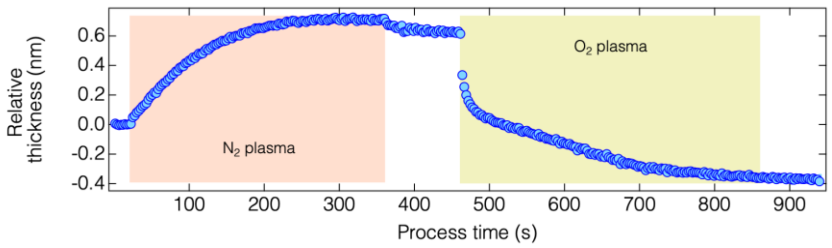

Fig. S2. An ALE cycle demonstrated for the self-limiting reactions during the N_2 and O_2 plasmas. The RF power for plasma discharges was set to 100 W. The bias power inputs for the N_2 and O_2 plasmas were set to 30 W and 0 W, respectively. The duration of the plasma discharges was determined based on the time required to achieve full saturation of the reaction.

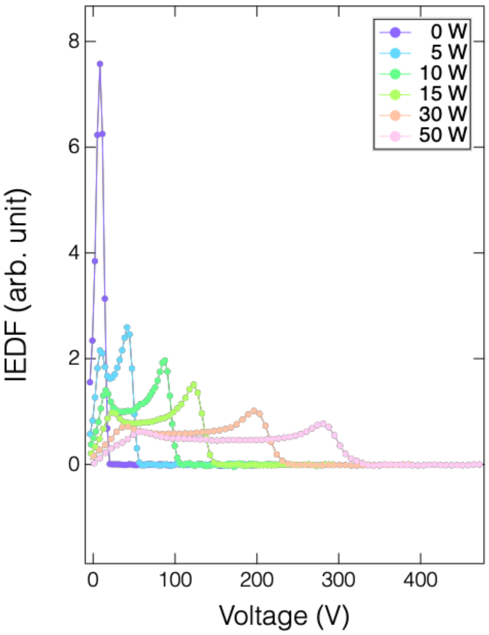

Fig. S3. Ion energy distribution function measured using retarding field energy analyzer (RFEA) during N_2 plasma treatment with various bias power inputs.

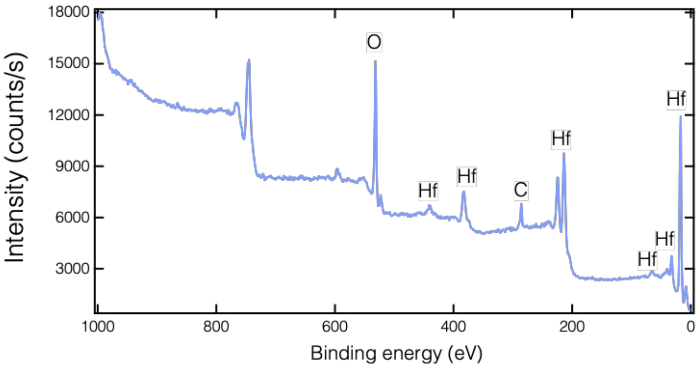

Fig. S4. XPS spectrum of HfO_2 film before ALE process.

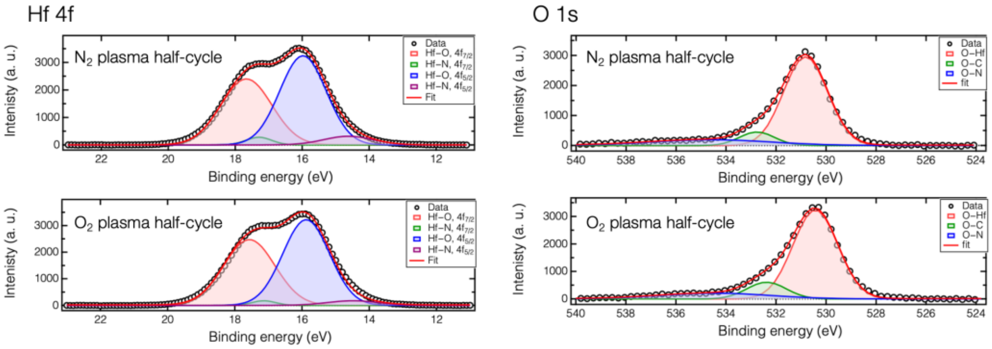

Fig. S5. Hafnium Hf 4f and oxygen O 1s XPS spectra of HfO_2 film treated with N_2 plasma in first half-cycle, followed by O_2 plasma in second half-cycle.

As shown in Fig. S5, for the Hf 4f spectra, although the components corresponding to Hf–N are more pronounced after the N_2 plasma half-cycle than after the O_2 plasma etching, the changes are relatively minor compared to the N 1s spectra. Furthermore, there is little difference between the spectra following the N_2 and O_2 half-cycles, which may be attributed to oxidation reactions occurring when the samples were exposed to air during transfer.

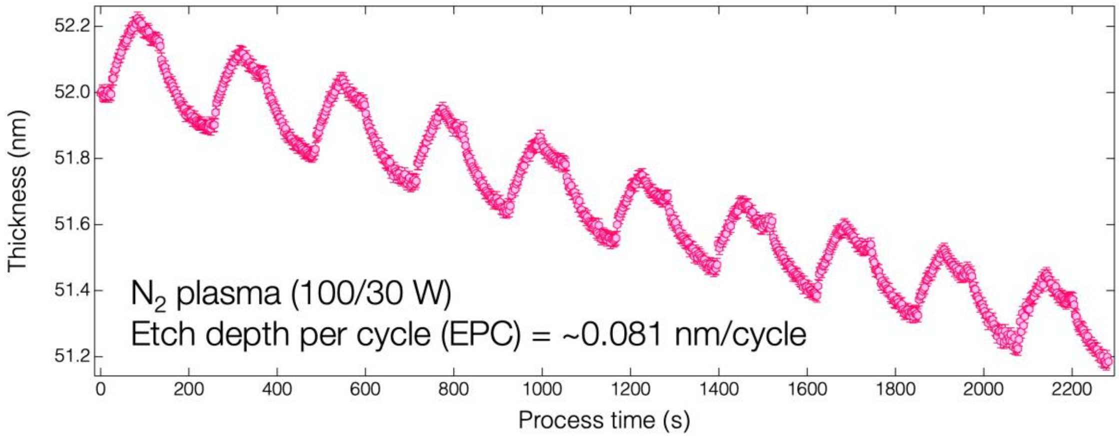

Fig. S6. Thickness variation for HfO_2 film prepared by high-power impulse magnetron sputtering over 10 cycles of ALE process.

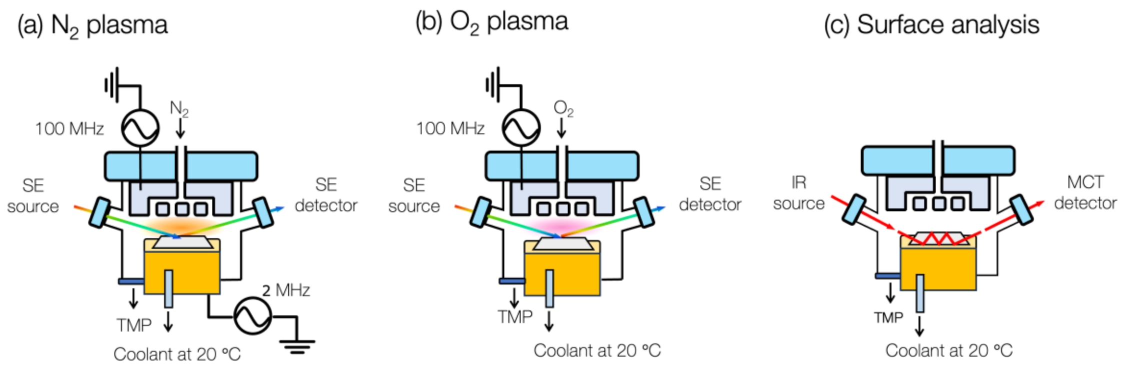

Fig. S7. Schematic of the homemade etching reactor used for the ALE process.
